# Evidence for efficient phosphorylation of EGFR and rapid endocytosis of phosphorylated EGFR via the early/late endocytic pathway in a gefitinib-sensitive non-small cell lung cancer cell line

**DOI:** 10.1186/1476-4598-7-42

**Published:** 2008-05-21

**Authors:** Yukio Nishimura, Kiyoko Yoshioka, Biborka Bereczky, Kazuyuki Itoh

**Affiliations:** 1 Division of Pharmaceutical Cell Biology, Graduate School of Pharmaceutical Sciences, Kyushu University, 3-1-1 Maidashi, Higashi-ku, Fukuoka 812-8582, Japan; 2 Department of Biology, Osaka Medical Center for Cancer and Cardiovascular Diseases, 1-3-2 Nakamichi, Higashinari-ku, Osaka 537-8511, Japan

## Abstract

Gefitinib (Iressa)–a specific inhibitor of epidermal growth factor receptor (EGFR) tyrosine kinase–has been shown to suppress the activation of EGFR signaling required for cell survival and proliferation in non-small cell lung cancer (NSCLC) cell lines. We recently provided novel evidence that gefitinib-sensitive PC9 cells show normal endocytosis of EGFR: internalized EGF-EGFR complexes were transported to late endosomes/lysosomes 15 min after EGF stimulation, and then degraded within the lysosomes. However, gefitinib-resistant QG56 cells showed internalized EGFR accumulation in early endosomes after 60 min of internalization, instead of its trafficking to lysosomes, indicating an aberration in some steps of EGF-EGFR trafficking from the early endosomes to late endosomes/lysosomes. Therefore, we postulate that impairment in some steps of EGF-EGFR trafficking from early endosomes to late endosomes/lysosomes might confer gefitinib-resistance in NSCLC cell lines. To further substantiate the detailed internalization mechanism of gefitinib-sensitive and gefitinib-resistant cells, using confocal immunofluorescence microscopy, we examined the endocytic trafficking of phosphorylated EGFR (pEGFR) in the absence or presence of gefitinib. In PC9 and QG56 cells without EGF stimulation, a large number of pEGFR-positive small vesicular structures not colocalized with late endosomes/lysosomes were spread throughout the cytoplasm, and some pEGFR staining was distributed in the nucleus. This implies a novel intracellular trafficking pathway for pEGFR from cytoplasmic vesicles to the nucleus. Furthermore, an aggregated vesicular structure of early endosomes was observed in the perinuclear region of QG56 cells; it was revealed to be associated with SNX1, originally identified as a protein that interacts with EGFR. Therefore, we confirmed our previous data that an aberration in some steps of EGF-EGFR trafficking from the early endosomes to late endosomes/lysosomes occurs in QG56 cells. Furthermore, in PC9 cells, efficient phosphorylation of EGFR and rapid internalization of pEGFR was observed at 3 min after EGF stimulation; these internalized pEGFR-positive vesicles were trafficked to late endosomes at 15 min, indicating rapid trafficking of EGF-pEGFR complexes from early to late endosomes in PC9 cells. Gefitinib treatment strongly reduced the phosphorylation level of EGFR, and subsequent endocytosis of EGFR was significantly suppressed in PC9 cells. In contrast, in QG56 cells, EGFR trafficking via the early endocytic pathway was basically impaired; therefore, gefitinib appeared to slightly suppress the internalization of pEGFR. Collectively, our data provide novel evidence that extensive impairment in pEGFR endocytosis via the early endocytic pathway might confer gefitinib-resistance in QG56 cells.

## Background

The epidermal growth factor receptor (EGFR) is a prototypical member of the ErbB family of tyrosine kinases and plays an important role in the pathogenesis of different tumors; therefore, therapies directed at inhibiting EGFR function have potential as anticancer treatments [1,2]. Each EGFR comprises an extracellular binding domain and a cytoplasmic domain with tyrosine kinase activity [3]. Following ligand binding, the EGFR is dimerized and the intracellular tyrosine kinase region is activated, causing receptor tyrosine autophosphorylation and transphosphorylation of another receptor monomer [4]. These events lead to the recruitment and phosphorylation of several intracellular substrates and the subsequent transmission of extracellular signals to the nucleus via an intracellular signaling network [4,5].

Gefitinib (Iressa, ZD1839) is a selective EGFR tyrosine kinase inhibitor that functions by competing with ATP for binding to the tyrosine kinase domain of the receptor, and it blocks the signal transduction pathways implicated in the proliferation and survival of cancer cells [6-9]. It has exhibited significant antitumor activity against a broad range of mouse tumor xenograft models in vivo [10] and tumor cell lines in vitro [11]. A recent in vitro study demonstrated that of the 9 non-small cell lung cancer (NSCLC) cell lines examined, the PC9 cell line was most sensitive to the effect of gefitinib when assayed under basal growth conditions for EGFR phosphorylation and activation of EGFR downstream effectors such as AKT and those in the ERK1/2 pathway, which are required for its survival and proliferation [11]. This suggests that the mechanism underlying the sensitivity of the EGFR pathway could be useful in predicting the potential effectiveness of gefitinib in NSCLC patients. Inefficient EGFR down regulation was observed in the gefitinib-resistant cell line QG56, whereas rapid down regulation occurred in the gefitinib-sensitive cell line PC9, wherein the cells were in the exponential phase of growth, suggesting that a different unknown down-regulation mechanism operates in each cell type.

For many years, the endocytosis of EGFR has served as a model for studying ligand-induced, receptor-mediated endocytosis. On EGF stimulation, EGF-EGFR complexes are internalized and transported via clathrin-coated vesicles to early endosomes. EGFR then recruits and phosphorylates signaling molecules, leading to the activation of an MAPK-signal transduction cascade–an important mechanism for regulating cell growth [12]. Once delivered to the lysosomes, EGF-EGFR complexes are degraded to cease intracellular EGFR signaling via endocytosis; this process is known as receptor down regulation. Therefore, endocytosis of EGF-EGFR complex is closely related with attenuation of intracellular EGFR signaling. With regard to the effect of gefitinib on the EGFR down-regulation pathway, we have recently examined the endocytosis of Texas red-EGF in the absence or presence of gefitinib in both PC9 and QG56 cell lines, and then assessed the endocytic pathway of internalized Texas red-EGF by using confocal immunofluorescence microscopy [13]. We found novel evidence that an aberration in some steps of EGF-EGFR trafficking from the early endosomes to the late endosomes/lysosomes does occur in the gefitinib-resistant human lung cancer cell line derived from NSCLC, whereas endocytosis of EGFR is normal in gefitinib-sensitive PC9 cells [13], suggesting that impairment in some steps of EGF-EGFR trafficking from early endosomes to late endosomes/lysosomes might confer gefitinib-resistance in NSCLC cell lines.

Based on these phenomena, in order to further investigate the relationship of EGFR signaling and EGFR endocytosis, we have now used confocal immunofluorescence microscopy to substantiate the detailed mechanisms for endocytosis of the ligand-induced activated form of EGFR, i.e., phosphorylated EGFR (pEGFR), via the early endosome/late endosome/lysosome endocytic pathway in both NSCLC cell lines, namely, the PC9 and QG56 cell lines. Here, we report novel data regarding the occurrence of rapid EGFR phosphorylation and endocytic delivery of pEGFR from early endosomes to late endosomes/lysosomes in PC9 cells after EGF stimulation; however, endocytosis of pEGFR is significantly perturbed via the early/late endocytic pathway in QG56 cells.

## Results

### Intracellular distribution of pEGFR in gefitinib-sensitive or gefitinib-resistant NSCLC cell lines

**Figure 1:**
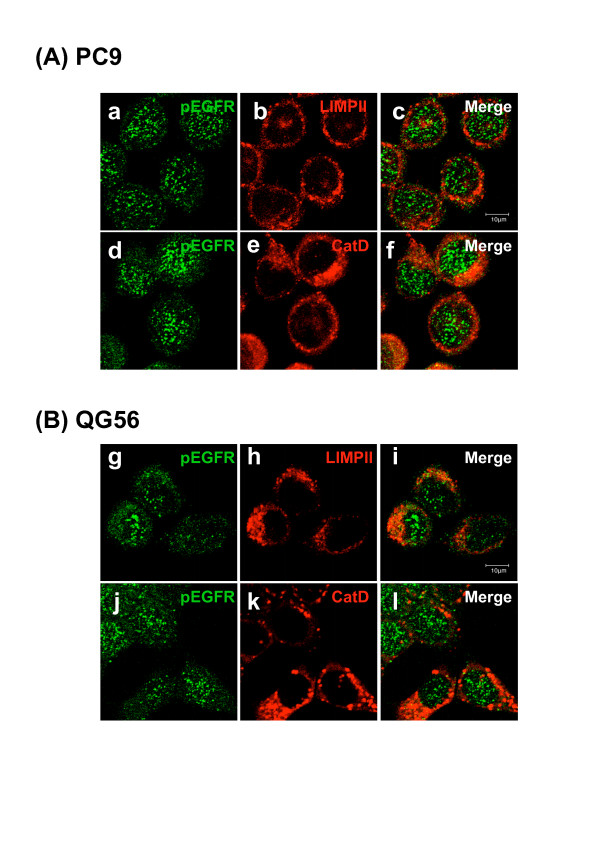
**Intracellular distribution of pEGFR in the NSCLC cell lines**. The gefitinib-sensitive NSCLC cell line, PC9 (A), or the gefitinib-resistant cell line, QG56 (B) was fixed and double-stained for LIMPII (red in b, h) or cathepsin D (red in e, k) and pEGFR (green) as described in the Materials section. Superimposed images of cathepsin D or LIMPII and pEGFR are shown in c, f, i, l. In both PC9 and QG56 cells, it is notable that pEGFR-positive small punctate vesicles are spreading in the cytoplasm and these vesicles are not colocalized with the LIMPII-, or cathepsin D-positive vesicular structures, and also pEGFR-positive punctate stainings are clearly seen in the nucleus. Bar, 10 μm.

In order to examine the intracellular distribution of pEGFR in the gefitinib-sensitive NSCLC cell line PC9 or the gefitinib-resistant cell line QG56, in the absence of EGF stimulation, each cell line was double-labeled either with antibodies specific to pEGFR or with those specific to cathepsin D and lysosomal integral membrane protein(LIMPII) (Fig. 1). We determined the intracellular distribution of late endosomes/lysosomes by using antibodies specific to lysosomal aspartic protease cathepsin D or LIMPII/lysosomal glycoprotein 85 (LGP85). These proteins are distributed within endocytic organelles and are at the highest concentration in the late endosomes/lysosomes, as observed for other lysosomal glycoproteins, namely, lysosomal associated membrane protein-1 (LAMP-1) and LAMP-2 [14-17].

In PC9 cells (A), it is notable that most pEGFRs were localized within small vesicular structures distributed throughout the cytoplasm, and it is clear that some punctate signals were found in the nucleus (a, d). The immunostaining pattern of pEGFR in QG56 cells (B) was similar to that in PC9 cells. Moreover, pEGFR staining was distributed in the cytosol and in the nucleus: some cytosolic pEGFR was stained diffusely, while no staining was observed in the plasma membrane (g, j). In both PC9 and QG56 cells, the small pEGFR-positive vesicular structures observed in large numbers were not costained with LIMPII or the cathepsin D antibody (c, f, i, l). These results indicate that pEGFR would be mainly distributed in the cytoplasmic vesicles and possibly in early endosomes; however, its distribution was also indicated in the nucleus at the steady-state level without EGF stimulation.

### Sorting nexin 1 (SNX1) is localized to the aggregated vesicular structures of early endocytic compartments in gefitinib-resistant NSCLC cells

It was reported previously that sorting nexin 1 (SNX1), originally identified as a protein that interacts with EGFR [18], is preferentially localized to early endosomes through its phospholipid-binding motif termed the phox homology (PX) domain [19]. It was also shown that overexpression of SNX1 caused enhanced EGFR degradation and that a deletion mutant of SNX1 blocked EGFR degradation but failed to inhibit receptor endocytosis [18,20]. Therefore, it is suggested that SNX1 plays a role in endosome-lysosome trafficking.

**Figure 2:**
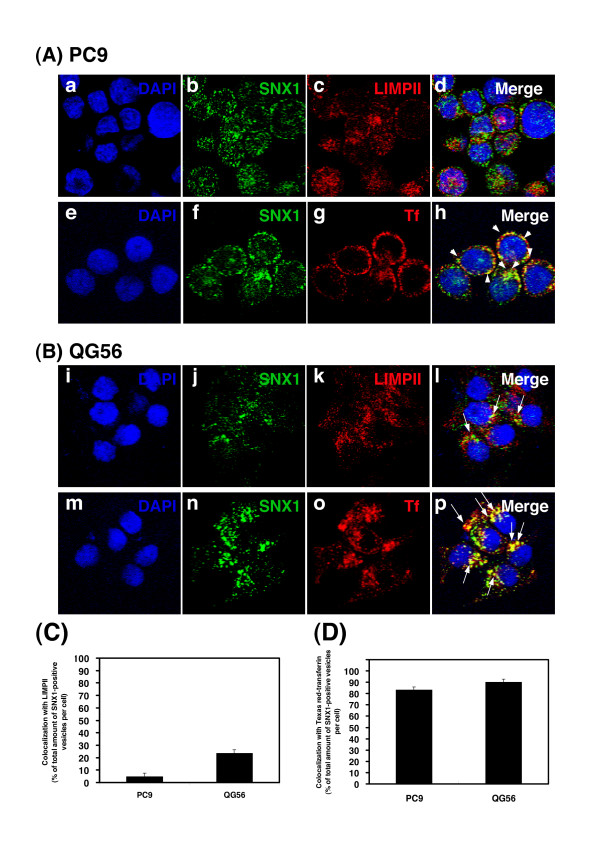
**Endocytosed Texas red-transferrin is downloaded into the SNX1-positive aggregated vesicular structure of early endosomes in the perinuclear region of gefitinib-resistant QG56 cells**. The PC9 cells (A), or the QG56 cells (B) were fixed and double-stained for SNX1 (green in b, j) and LIMPII (red in c, k) as described in the Materials section. Superimposed images of SNX1 and LIMPII are shown in d, l. Each cell line was stained with DAPI (blue) to reveal nuclei. The merged confocal images as yellow color were quantified and presented as the percentage of total amounts of SNX1-positive vesicles per cell in C. The error bar denotes SD. In PC9 cells (A), SNX1-positive small vesicles are not colocalized with LIMPII-positive vesicles (d), however, in QG56 cells (B), part of LIMPII-positive vesicles colocalized with SNX1-positive early endosomes is seen in the perinuclear region (l). Furthermore, superimposed images of SNX1 and the internalized Texas red-transferrin in the PC9 cells and the QG56 cells are shown in h and p, respectively. The merged confocal images as yellow color as indicated by white arrowheads (h) or white arrows (p) were quantified and presented as the percentage of total amounts of SNX1-positive vesicles per cell in D. Note that SNX1-positive early endosomes form large aggregated vesicular structures in the perinuclear region (p) in QG56 cells, and that these aggregated structures are overlapped with Texas red-transferrin; however no aggregated vesicles are seen in PC9 cells (h).

In the present study, to investigate the intracellular distribution of SNX1 with endocytosed transferrin–a marker of early endosomes in NSCLC cell lines–PC9 or QG56 cells were allowed to internalize Texas red-labeled transferrin for 20 min. After transferrin binds to its receptor on the cell surface, it is internalized via clathrin-coated vesicles and is subsequently delivered to the early endosomes. Confocal immunofluorescence microscopy studies revealed that endogenous SNX1 was distributed primarily to punctate vesicles and that it showed considerable overlap with endocytosed transferrin in the cytoplasm of PC9 cells (Fig. 2A, h). However, it was also shown that SNX1 staining did not overlap with late endosomes/lysosomes labeled by the LIMPII antibody (Fig. 2A, d). In contrast, SNX1 was distributed in the aggregated vesicular structures in the perinuclear region of QG56 cells, and SNX1 staining overlapped with Texas red-transferrin-positive early endosomes (Fig. 2B, p). Interestingly, a part of SNX1-positive aggregated vesicles were also colocalized with late endosomes labeled with the LIMPII antibody (Fig. 2B, l), therefore, indicating that membrane trafficking of EGFR between early endosomes and late endosomes might be considerably suppressed in QG56 cells. However, it was revealed to be normal in PC9 cells. Furthermore, quantitative analysis was performed to determine the amounts of SNX1 that colocalized with LIMPII (Fig. 2C) or with endocytosed Texas red-transferrin (Fig. 2D). These results confirm the presence of an aberration in the early endosomes of QG56 cells.

### Efficient phosphorylation of EGFR and rapid endocytosis of pEGFR via the early/late endocytic pathway in the gefitinib-sensitive NSCLC cell line

Receptor tyrosine kinases play important roles in cell growth, survival, migration, and differentiation. Ligand-induced activation of receptor tyrosine kinases leads to the assembly of signaling protein complexes and subsequent activation of downstream signaling pathways [12]. The endocytosed receptors then undergo a sorting process that determines the fate of the receptor and signal intensity. These receptors are targeted to the lysosomes for degradation–a process that terminates receptor signaling [21].

**Figure 3:**
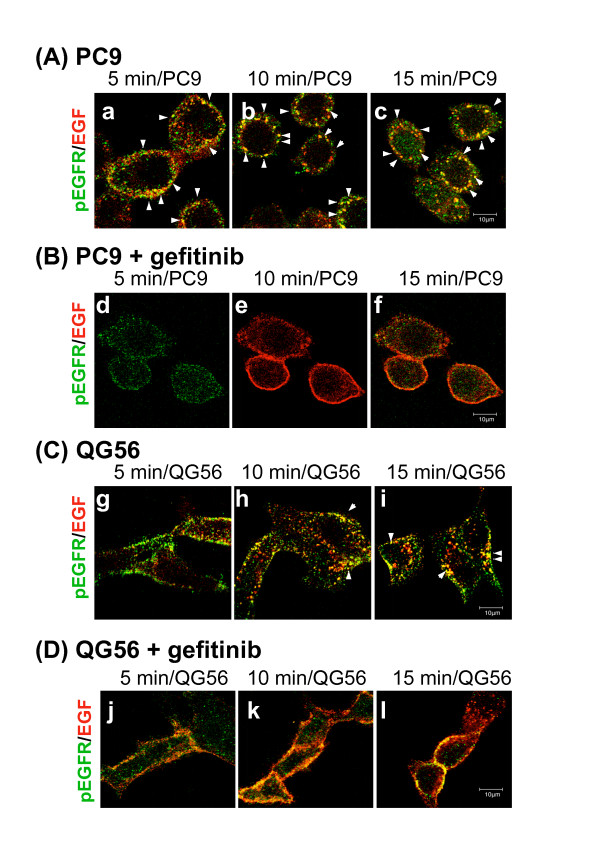
**Evidence for a rapid endocytosis of ligand-induced pEGFR in the gefitinib-sensitive PC9 cells, but for an inefficient endocytic traffic of EGF-pEGFR in the gefitinib-resisitant QG56 cells**. The PC9 (A, B) or QG56 (C, D) cells were incubated in the absence (A, C) or presence (B, D) of gefitinib at 37°C with Texas red-EGF for 5, 10, or 15 min, and cells were fixed and double-stained for pEGFR (green) as described in the Materials section. Superimposed images of pEGFR and Texas red-EGF are shown. The white arrowheads indicate the colocalization of the pEGFR-positive vesicles and Texas red-EGF-positive vesicular structures. It is notable that rapid endocytosis of EGF-EGFR occurs in PC9 cells, since large amounts of pEGFR-positive small vesicles co-stained with Texas red-EGF appear in the cytoplasm after 5 min incubation (a) and these co-stained vesicles are increased at 15 min (b, c). By contrast in QG 56 cells, pEGFR stainings are mostly associated with plasma membrane even after 15 min incubation (i). Further, gefitinib significantly suppresses phosphorylation of EGFR in NSCLC cell lines and amount of pEGFR stainings are considerably reduced during the incubation (d, e, f, j, k, l). Bar, 10 μm.

In this study, we have substantiated the detailed mechanisms for endocytosis of the ligand-induced activated form of EGFR, i.e., pEGFR, via the early/late endocytic pathway in both NSCLC cell lines, i.e., PC9 and QG56 cell lines. To clarify EGFR internalization for each cell line, we monitored the uptake of Texas red-conjugated EGF with time. To minimize the involvement of recycling and/or lysosomal degradation of the internalized EGFR, we quantified Texas red-EGF uptake in each transfectant for various time periods up to 15 min. The cells were incubated with Texas red-EGF in the absence (A, C) or presence (B, D) of gefitinib at 37°C for 5, 10, and 15 min. Confocal immunofluorescence microscopy was then used to assess the distribution of internalized Texas red-EGF and endocytosed vesicles stained with the anti-pEGFR antibody (Fig. 3).

In the gefitinib-sensitive cell line PC9, efficient internalization of pEGFR was observed after 5 min of internalization, since large amounts of pEGFR staining was observed to have colocalized with Texas red-EGF-positive small endocytic vesicles, presumably early endosomes, in the vicinity of the plasma membrane (a). Moreover, these vesicles costained with internalized pEGFR and Texas red-EGF were maturated and distributed in the periphery of the nucleus; they showed a gradual increase in size until 15 min of incubation (b, c). These pEGFR- and EGFR-costained vesicles are considered to be late endosomes/lysosomes. This therefore indicates rapid delivery of the endocytosed EGF-EGFR complex from the early endosomes to the late endosomes, after EGF stimulation in PC9 cells [13].

In contrast, in the gefitinib-resistant cell line QG56, internalization of Texas red-EGF was suppressed (Fig. 3C). After 5 min of incubation, large amounts of pEGFR-positive vesicular structures were associated with the plasma membrane, and internalization of Texas red-EGF was not observed in the cell. Some pEGFR-positive vesicles overlapping with Texas red-EGF staining were revealed to have accumulated as aggregated structures in the vicinity of the plasma membrane at 15 min of incubation (i). These data indicate that EGF stimulation induces phosphorylation of EGFR in the plasma membrane of QG56 cells; however, internalization of the pEGFR-EGF complex from the plasma membrane to endocytic vacuoles is fairly suppressed in this cell line. This is consistent with our previously reported novel evidence that in QG56 cells, the endocytic machinery of EGFR is basically impaired at the level of the early endocytic pathway [13].

Gefitinib is an active EGFR tyrosine kinase inhibitor that competes for the ATP-binding site in the cytoplasmic tail, thus inhibiting EGFR activation and the transduction of post-receptor signaling pathways. Using confocal immunofluorescence microscopy, we have recently demonstrated that in PC9 cells, gefitinib significantly inhibited the efficient internalization rate of Texas red-EGF in the early stage of endocytosis, from the plasma membrane to the early endosomes; furthermore, it was also indicated that the suppressive effect of gefitinib on the endocytosis of EGFR proved to be much stronger in PC9 cells than in QG56 cells [13].

In the present study, to further substantiate the effect of gefitinib on the EGFR down-regulation pathway and to understand the internalization mechanism of PC9 cells or QG56 cells in detail, we examined the effect of gefitinib on the phosphorylation of EGFR and the subsequent internalization of pEGFR in the presence of gefitinib in each cell line for various time periods upto 15 min. Confocal immunofluorescence microscopy was used to assess the distribution of internalized Texas red-EGF and intracellular vesicles stained with the anti-pEGFR antibody. The results revealed that the gefitinib treatment strongly reduced the phosphorylation level of EGFR and that the endocytosis of EGFR was significantly suppressed in PC9 cells (Fig. 3B). Ever after 15 min of internalization, most of the Texas red-EGF remained associated with the plasma membrane of gefitinib-treated PC9 cells instead of being trafficked to the early endosomes (Fig. 3B, f). Similarly, the suppression of EGFR phosphorylation was observed in QG56 cells; in most of the Texas red-EGF-stained cells, no internalization of Texas red-EGF staining was observed even after 15 min of incubation, since it remained attached to the plasma membrane (Fig. 3D, l). These results indicate that in PC9 cells, gefitinib significantly inhibits the efficient phosphorylation of EGFR and rapid internalization of pEGFR in the early stage of endocytosis, from the plasma membrane to the early endosomes. Further, the suppressive effect of gefitinib on the endocytosis of pEGFR proved to be much stronger in PC9 cells than in QG56 cells, since pEGFR trafficking via the early endocytic pathway is basically perturbed in QG56 cells; however, the pEGFR endocytic pathway is normal in PC9 cells.

### Phosphorylated EGFR is rapidly endocytosed and trafficked to late endosomes/lysosomes in gefitinib-sensitive cell lines

To clarify pEGFR internalization, confluent NSCLC cell lines were cultured in serum-free medium for 3 h, and then EGFR phosphorylation was induced by incubation with EGF (100 ng/ml) for 15 min on ice in a binding medium (1 mg/ml bovine serum albumin (BSA) in RPMI medium). The cells were then rinsed with ice-cold phosphate-buffered saline (PBS), incubated in the presence of Texas red-transferrin in a prewarmed medium, and chased at 37°C for 3, 6, and 15 min.

**Figure 4:**
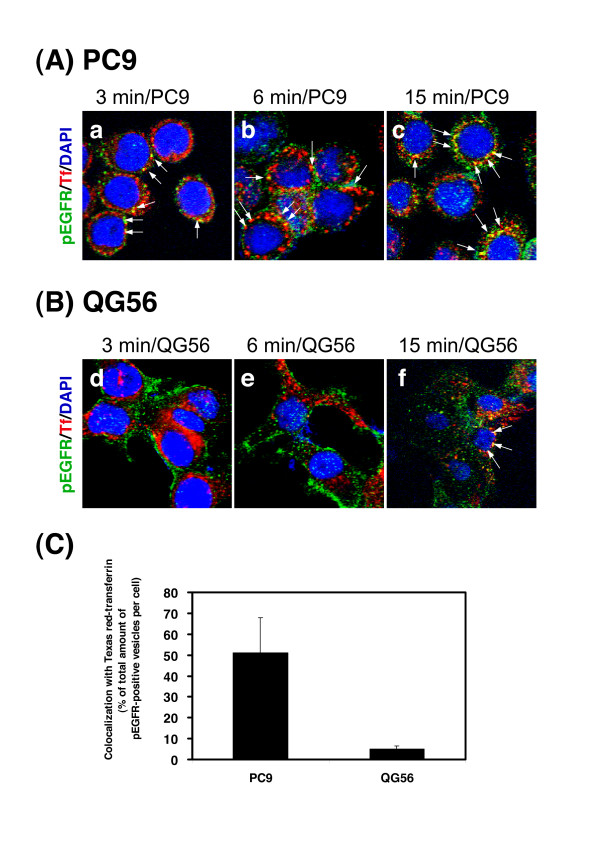
**Evidence for an efficient phosphorylation of EGFR and rapid delivery of pEGFR into the early endosomes after EGF stimulation in PC9 cells**. The PC9 (A) or QG56 (B) cells stimulated with EGF for 15 min on ice were further incubated at 37°C with Texas red-transferrin (red) for 3, 6, or 15 min, and cells were fixed and double-stained for pEGFR (green) as described in the Materials section. Superimposed images of pEGFR and Texas red-transferrin are shown. Each cell line was stained with DAPI (blue) to reveal nuclei. The white arrows indicate the colocalized early endosomal vesicular structures positive for pEGFR and Texas red-transferrin. The merged confocal images as yellow color as indicated by white arrows (a, d) at 3 min incubation were quantified and presented as the percentage of total amounts of pEGFR-positive vesicles per cell in D. In PC9 cells (A), ligand-induced EGFR phosphorylation occurs efficiently in early endosomes or in plasma membrane (a, b, c). By contrast, only small fraction of pEGFR staining associated with early endosomal vesicles is seen in the cytoplasm of QG56 cells even after 15 min incubation (f).

Using confocal immunofluorescence microscopy, we then assessed the intracellular distribution of pEGFR and internalized Texas red-transferrin, an endocytic marker (Fig. 4). We previously reported that in NSCLC cell lines or in human breast cancer cell lines, internalized transferrin is predominantly distributed in the form of small punctate structures in the perinuclear and peripheral regions, presumably representing recycling endosomes and sorting endosomes, respectively [13,22-24]. The gefitinib-sensitive cell line PC9 showed rapid internalization of pEGFR since pEGFR-positive vesicular structures were observed in the cell at 3 min of internalization; moreover, these pEGFR-positive vesicles were colocalized with transferrin receptor-positive early endosomes (Fig. 4A, a), indicating rapid phosphorylation of EGFR and its efficient delivery to early endosomes after EGF stimulation. After 15 min of internalization, an increasing number of pEGFR-positive vesicular structures costained with endocytosed Texas red-transferrin was observed in the vicinity of the nucleus (Fig. 4A, c).

In contrast, in the gefitinib-resistant cell line QG56, internalization of pEGFR was suppressed (Fig. 4B). Even after 15 min of internalization, only a small number of pEGFR-positive vesicles associated with the internalized Texas red-transferrin staining were observed in the perinuclear region of QG56 cells (Fig. 4B, f). These data indicate that in QG56 cells, an aberration in pEGFR endocytosis occurs via the early/late endocytic pathway and the delivery of pEGFR from the early endosomes to late endosomes/lysosomes is also considerably perturbed. Quantitative analysis was performed to determine the amount of Texas red-transferrin-positive early endosomal markers (Fig. 4C) that colocalized with the endocytosed pEGFR at 3 min of internalization; it was confirmed that rapid delivery of pEGFR to early endosomes is observed to a greater extent in PC9 cells than in QG56 cells.

**Figure 5:**
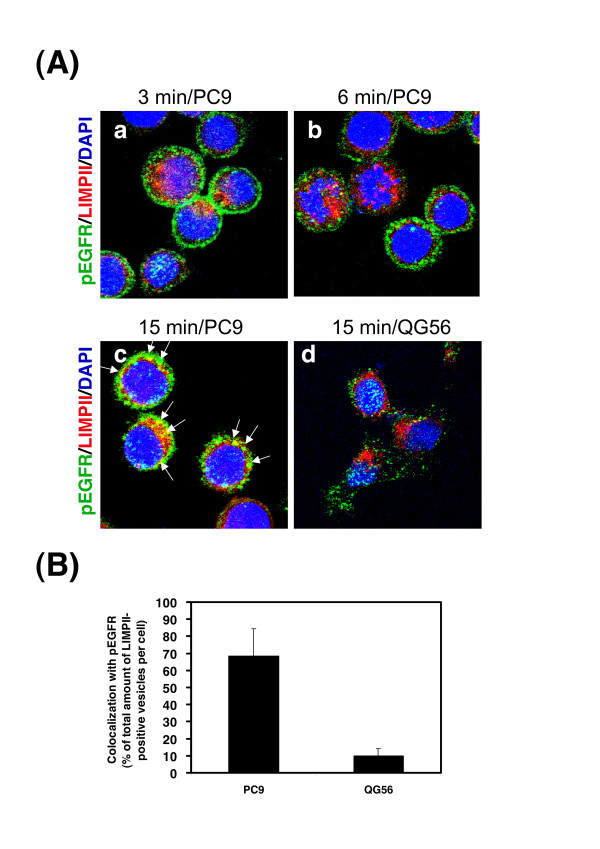
**Identification for an efficient pEGFR trafficking via early/late endocytic pathway in PC9 cells**. The PC9 or QG56 cells were preincubated with EGF for 15 min on ice and then cell were chased at 37°C for 3, 6, or 15 min, and cells were fixed and double-stained for pEGFR (green) or LIMPII (red) as described in the Materials section. Superimposed images of pEGFR and LIMPII are shown. Each cell line was stained with DAPI (blue) to reveal nuclei. The white arrows indicate the colocalized LIMPII-positive late endosomes/lysosomes and pEGFR-positive cytoplasmic vesicular structures. The merged confocal images as yellow color as indicated by white arrows (c, d) at 15 min incubation were quantified and presented as the percentage of total amounts of LIMPII-positive vesicles per cell in B. It is notable that an efficient trafficking of pEGFR to late endosomes is seen in PC9 cells (a, b, c), but is not observed in QG56 cells (d).

To further examine pEGFR internalization, using confocal immunofluorescence microscopy, the intracellular fate of pEGFR that colocalized with the late endosome/lysosome marker stained with the LIMPII antibody was monitored for various time periods upto 15 min by using each cell line. As shown in Fig. 5A, in PC9 cells, an increasing number of pEGFR-positive vesicles colocalized with LIMPII-positive late endosomes/lysosomes were observed in the cytoplasm at 15 min of internalization (Fig. 5A, c); however, in QG56 cells, no pEGFR-positive vesicular structures overlapping with LIMPII-positive late endosomes/lysosomes were observed (Fig. 5A, d). We have previously reported that the EGF-EGFR complex associated with the plasma membrane is efficiently endocytosed and translocated to LIMPII-positive late endosomes/lysosomes at 15 min after EGF stimulation in PC9 cells  [13]; therefore, our present data showing the efficient trafficking of pEGFR from the plasma membrane via early endosomes to late endosomes within 15 min of EGF stimulation is consistent with that reported previously. These results further confirm that ligand-induced pEGFR endocytosis operates normally in PC9 cells, but pEGFR trafficking is attenuated at the level of the early endocytic pathway in QG56 cells. Quantitative analysis for determining the amounts of LIMPII-positive late endosome/lysosome markers (Fig. 5B) that colocalized with the endocytosed pEGFR at 15 min of internalization confirmed the efficient trafficking of pEGFR to late endosomes in PC9 cells and attenuation of endocytic vesicular trafficking in QG56 cells.

## Discussion

In the present study, using confocal immunofluorescence microscopy, we demonstrated that in PC9 cells, efficient phosphorylation of EGFR and rapid internalization of pEGFR occurred at 3 min after EGF stimulation, since large amounts of small pEGFR-positive punctate vesicles were colocalized with the internalized Texas red-transferrin-positive early endosomes in the vicinity of the plasma membrane. These internalized pEGFR-positive vesicles were maturated and subsequently trafficked to LIMPII-positive late endosomes/lysosomes in the periphery of nucleus, along with gradual increases in its size at 15 min after EGF stimulation. These results indicate that efficient membrane trafficking of the EGF-pEGFR complex from early endosomes to late endosomes occurs in PC9 cells, and also suggest that ligand-induced EGFR signaling might operate in the early endosomes/late endosomes via the endocytic pathway. Furthermore, in the gefitinib-treated PC9 cells, endocytic trafficking of EGFR was significantly impaired and unphosphorylated EGFR remained associated with the plasma membrane. Therefore, the suppressive effect of gefitinib on the phosphorylation level of EGFR was demonstrated. In contrast, internalization of pEGFR was basically suppressed in QG56 cells; therefore, the inhibitory effect of gefitinib on pEGFR trafficking is limited. Collectively, our data indicate that an aberration in pEGFR endocytosis occurs via the early/late endocytic pathway and that the delivery of pEGFR from the early endosomes to the late endosomes/ysosomes is considerably perturbed in QG56 cells. Based on these findings, we postulate that efficient endocytosis of ligand-induced EGFR is closely related to EGFR-tyrosine kinase inhibitor sensitivity of human lung cancer cell lines.

We further detected clear nuclear staining of pEGFR in PC9 and QG56 cells, although considerable amounts of pEGFR were distributed in small cytoplasmic punctate vesicles. Since we recently demonstrated that in PC9 cells, most EGFR is localized in the plasma membrane and in LIMPII-positive swollen vacuoles, i.e., late endosomes [13], it is interesting to note that a part of pEGFR is already localized in the nucleus even in the absence of EGF stimulation. These results imply that pEGFR might be translocated into the nucleus through the conventional nuclear importing system associated with the nuclear pore complex (that is, the Ran/importin pathway), and it might then operate as a transcription factor. In fact, it has recently been reported that nuclear localization of EGFR is detected in the highly proliferating state of human cancer tissues in vivo and human breast cancer cell lines in vitro, supporting the close correlation between nuclear EGFR and tumor tissues with high proliferation [25-27]. It was also shown that nuclear EGFR levels were increased on treatment with EGF and that the EGFR which accumulated in the nucleus was highly tyrosine phosphorylated; it was further demonstrated that nuclear EGFR acts as a transcription factor for activating gene expression of cyclin D1, a well-known cell growth-promoting factor [26]. Therefore, further studies with respect to the functions of these cell growth nuclear receptors should be conducted to create new avenues in the field of receptor signaling.

With regard to the endocytosis of EGFR via the early endocytic pathway in gefitinib-resistant cell lines, for QG56 cells, we reported novel evidence regarding the accumulation of internalized EGF-EGFR in the early endosomes, instead of its trafficking to the lysosomes; this evidence suggests that the endocytic machinery of EGFR might be considerably impaired at the level of the early/late endosomes [13]. To further substantiate this, we examined the intracellular distribution of SNX1 along with endocytosed transferrin in NSCLC cell lines. SNX1 is a mammalian homologue of yeast Vps5p, which recognizes the lysosomal targeting code of EGFR and participates in lysosomal trafficking of the receptor [18,28]; SNX1 is preferentially localized to early endosomes through its phospholipid-binding motif termed the PX domain [19]. In the present study, using confocal immunofluorescence microscopy, we demonstrated novel evidence that in QG56 cells, early endosomes labeled with endocytosed Texas red-transferrin formed an aggregated vesicular structure distributed in the perinuclear region and SNX1 distribution overlapped with these aggregated early endosomal vesicles. Surprisingly, a part of SNX1-positive aggregates was also colocalized with late endosomes labeled with the LIMPII antibody, implying that membrane trafficking of EGFR from the early endosomes to late endosomes might be significantly impaired in QG56 cells; however, no such accumulation was noted in PC9 cells. Therefore, we speculate that impairment of SNX1 trafficking might cause the perturbation of EGFR endocytosis, which then leads to the acquisition of gefitinib-resistance in NSCLC cell lines.

It has been previously reported that gefitinib-sensitive lung tumors were found to express EGFR variants with a higher sensitivity for the drug than the wild-type receptor [29,30]. Since it has been known that PC9 cells express EGFR mutant variants and QG56 cells express wild-type EGFR [11], it can be assumed that the drug suppresses the endocytosis of EGFR more strongly in gefitinib-sensitive PC9 cells than in gefitinib-resistant QG56 cells. Recently, cellular targets of gefitinib were identified in HeLa cells by a proteomic method in which gefitinib could interact with more than 20 previously unknown kinase targets [31]. They further found novel evidence that gefitinib could interact with the serine/threonine kinase, cyclin G-associated kinase (GAK), which has been recently indicated to act as a negative regulator of EGFR signaling [32], thereby proposing that the gefitinib-mediated inactivation of a negative regulator would antagonize the inhibitory effect of the drug on EGFR signaling. It is important to further analyze the mechanism by which the interaction of EGFR and other cellular proteins with gefitinib might regulate the process of EGFR down regulation in NSCLC cell lines. Further analysis of the endocytosis machinery in gefitinib-sensitive or gefitinib-resistant NSCLC cell lines could be useful for clarifying the effectiveness of gefitinib.

## Conclusion

We found novel evidence for efficient EGFR phosphorylation and rapid endocytic delivery of pEGFR from plasma membrane to early endosomes/late endosomes/lysosomes in the gefitinib-sensitive cell line PC9 cells after EGF stimulation; however, pEGFR trafficking via the early endocytic pathway was basically impaired in the gefitinib-resistant cell line QG56 cells, since internalized pEGFR was accumulated in the aggregated vesicular structures of early endosomes associated with SNX1 instead of its trafficking to late endosomes/lysosomes. Therefore, we suggest that extensive impairment in pEGFR endocytosis via the early endocytic pathway might confer gefitinib-resistance in QG56 cells. Thus impairment of protein function such as SNX1 regulating EGFR trafficking in the early endocytic pathway might cause the perturbation of EGFR endocytosis, which then leads to the acquisition of gefitinib-resistance in NSCLC cell lines.

## Methods

### Materials

Gefitinib was provided from AstraZeneca (Macclesfield, United Kingdom). Texas red-labeled transferrin, Texas red-labeled EGF, and SlowFade anti-fade reagent were purchased from Molecular Probes (Eugene, OR, USA). DAPI was obtained from Sigma (St. Louis, MO, USA). Recombinant human EGF was purchased from PeproTech (London, United Kingdom). Other chemicals were of reagent grade and were obtained from commercial sources.

### Cell culture

Cell lines PC9 and QG56 (Kyushu Cancer Center, Fukuoka, Japan) were cultured in RPMI supplemented with 10% fetal bovine serum (FBS). Cells were maintained under standard cell culture conditions at 37°C and 5% CO_2 _in a humid environment.

### Antibodies

Alexa 488-labeled goat anti-mouse and goat anti-rabbit secondary antibodies, Texas red-labeled human transferrin, and Texas red-labeled EGF were obtained from Molecular Probes (Eugene, OR, USA). Normal goat serum was purchased from Sigma (St. Louis, MO, USA). Antisera were raised in rabbits (New Zealand white male) against the mature form of rat liver lysosomal cathepsin D [33,34] and the native form of LIMPII/LGP85 [15] as described previously. Anti-cathepsin D or anti-LIMPII IgG was affinity-purified by protein A Sepharose CL-4B (Sigma), followed by immunoaffinity chromatography using antigen-conjugated Sepharose 4B. A mouse monoclonal anti-pEGFR was obtained from DakoCytomation (Denmark) and BD Biosciences (San Jose, CA, USA). Mouse monoclonal antibody to SNX1 was purchased from BD Biosciences (San Jose, CA, USA).

### Immunofluorescence microscopy

Immunofluorescence microscopy was described previously [13,22-24]. In brief, cells were grown for 2 days on glass coverslips in 6-well plates in RPMI with 10% fetal bovine serum. Cells were fixed with 3.7% formaldehyde in phosphate-buffered saline (PBS), pH 7.4, permeabilized in PBS containing 0.1% saponin. After washing with PBS, cells were blocked with PBS-10% normal goat serum. All subsequent antibody and wash solutions contained 0.1% saponin. The PC9 or QG56 cells were incubated with specific primary antibodies (rabbit anti-cathepsin D and anti-LIMPII IgGs, mouse anti-pEGFR mAb, or mouse anti-SNX1 mAb), for 1 h, followed by washes with PBS containing 0.1% saponin and incubation for 1 h with the secondary antibodies at 20 μg/ml. Each cell line was stained with DAPI to reveal nuclei. To label early endosomes, cells were incubated with RPMI without FBS for 3 h at 37°C followed by 20 min incubation in culture medium containing Texas red-conjugated transferrin, and then cells were fixed and stained for SNX1. Controls for antibody specificity were either preimmune serum (rabbit or mouse) or omission of the primary antibodies. To follow the endocytic pathway and determine the intracellular fate of internalized labeled ligand, the uptake of Texas red-conjugated-EGF by the cells was measured. The PC9 or QG56 cells were starved for 3 h with RPMI without FBS at 37°C. The serum-starved cells were preincubated for 3 h in the absence or presence of 0.1 μM gefitinib before incubation with Texas red-EGF (100 ng/ml) at 37°C for 5, 10, and 15 min, and then the cells were fixed and stained for pEGFR. The distribution of the labeled proteins was analyzed by confocal immunofluorescence microscopy of the fixed cells. In some cases, PC9 or QG56 cells were starved for 3 h with RPMI without FBS at 37°C and then the phosphorylation of the EGFR was induced with EGF (100 ng/ml) for 15 min on ice in binding medium (1 mg/ml BSA in RPMI medium). The cell were then rinsed with ice-cold PBS, incubated in the presence of Texas red-transferrin in prewarmed medium, and chased at 37°C for 3, 6, and 15 min. Slides were mounted with SlowFade anti-fade reagent and observed on a Zeiss LSM 510 META confocal laser scanning microscope (Carl Zeiss, Oberkochen, Germany), equipped with krypton/argon laser sources. For quantification of colocalization between Texas red-EGF and pEGFR, between Texas red-transferrin and SNX1, between Texas red-transferrin and pEGFR, or between LIMPII and pEGFR, merged images as yellow color were quantified and presented as the percentage of total amounts of SNX1-, pEGFR- or LIMPII-positive vesicles per cell.

## Abbreviations

EGFR: Epidermal growth factor receptor; pEGFR: Phosphorylated epidermal growth factor receptor; NSCLC: Non-small cell lung cancer cell lines; LIMPII: Lysosomal integral membrane protein II; SNX1: Sorting nexin 1

## Competing interests

The authors declare that they have no competing interests.

## Authors' contributions

YN conceived the study, carried out experimental work and participated in the interpretation of the results and wrote the paper, KY and BB performed experiments and analyzed the data, KY and KI provided supports for the work and also provided critical comments in the drafting of the manuscript. All authors approved the final version of the manuscript.
